# Influence of Injection Molding Parameters and Distance from Gate on the Mechanical Properties of Injection-Molded Polypropylene

**DOI:** 10.3390/polym17081012

**Published:** 2025-04-09

**Authors:** Klara Fucikova, Martin Ovsik, Adam Cesnek, Adam Pis, Jiri Vanek, Michal Stanek

**Affiliations:** Faculty of Technology, Tomas Bata University in Zlin, Vavreckova 5669, 760 01 Zlin, Czech Republic; k_fucikova@utb.cz (K.F.); a_cesnek@utb.cz (A.C.); a_pis@utb.cz (A.P.); vanek@utb.cz (J.V.); stanek@utb.cz (M.S.)

**Keywords:** polypropylene, injection molding, injection pressure, mold temperature, mechanical properties, indentation hardness

## Abstract

This publication deals with the study of the mechanical properties of injection-molded polypropylene parts depending on the process parameters and the distance from the gate location in which the mechanical properties were investigated. Due to the fact that the mechanical properties of injection-molded parts are not the same at all locations, this research was designed to investigate the inhomogeneity of the properties of injection-molded parts along the length of the product. The inhomogeneity is affected by various influences, including distance from the sprue mouth, melt and mold temperature, injection pressure, crystal structure, and others. It was demonstrated that mechanical properties are not uniform over the entire injected product. Contrary to popular belief, mechanical properties can vary along the flow length due to uneven cooling and process parameters. Injection pressure and mold temperature significantly affect the mechanical properties of the injection-molded parts. The limiting injection pressure is 40 MPa and the mold temperature is 40 °C. The difference in individual spots in an injected article was up to 37%. Changes in mechanical properties are closely related to changes in morphology (crystallinity measured by DSC) caused by different injection molding process parameters. As is evident from the aforementioned results, the possible benefits of this work for injection molding of polymer products are apparent. Suitably chosen gate location, surface of the cavity, and process parameters can ensure targeted improvement of mechanical properties in stressed parts of a product.

## 1. Introduction

Injection molding technology is one of the most widely used methods of producing a part from a polymer. The influence of injection molding parameters and the distance from the gate is a critical area of study in materials science, particularly in the production of polymer components. During the production cycle, thermal and mechanical influences act simultaneously on the polymer and affect the final properties of the product. Injection molding, a widely utilized manufacturing process, involves the injection of molten material into a mold, where it cools and solidifies into the desired shape. The parameters involved in this process, such as injection speed, temperature, and pressure, significantly affect the final product’s mechanical properties, dimensional accuracy, and overall quality.

The resulting mechanical, physical, and optical properties of the injection-molded part are closely related to the microstructure formed on the wall and also in the core of the part. The different morphology of the core surface has been studied in many papers [1,2,3,4,5,6,7]. It is referred to as skin–core morphology and is observable by polarized light microscopy. The formation of two to five layers has been demonstrated in injection-molded parts made of semicrystalline polymers [8,9,10].

The appropriate setting of process parameters in injection molding technology is very important to maintain the stability of the production process, but also in terms of the final properties of the manufactured parts. The most important parameters that significantly affect the injection process are injection pressure, injection speed, holding pressure, duration of holding pressure, melt temperature, and mold temperature. The individual process parameters set act simultaneously and influence each other; changing one parameter affects the other parameters [1].

Several publications deal with the influence of process conditions on the mechanical properties of the product. The study by Wang et al. [11] focused mainly on the effect of injection speed on the mechanical properties of a microinjected polypropylene sample. The study found that there is an increase in hardness with increasing injection speed and that this parameter has a greater effect on the properties perpendicular to the melt flow direction than in the melt flow direction. A similar issue was also addressed by Głogowska et al. [12], who injected samples under different process conditions and subsequently grinded and re-injected these samples from the crush. The mechanical properties were measured on these samples.

Sykutera et al. [13] investigated the effect of process conditions on the viscosity of polymer melt measured directly in the injection mold cavity, providing realistic values directly from the injection molding process. Other studies [14,15,16,17] investigated the effect of different process conditions (pressure and temperature) on the final melt flow length.

For instance, studies have shown that variations in injection speed can lead to differences in the molecular orientation of the polymer, which can impact the mechanical properties of the final product [18,19,20]. Additionally, the temperature profile during the injection process is crucial; higher melt temperatures can enhance flowability but may also lead to thermal degradation of the polymer if not controlled properly [21,22].

Furthermore, the cooling rate is a critical parameter that affects the crystallization and final properties of the molded part. The cooling rate can be influenced by the mold temperature, the design of the cooling channels, and the distance from the gate. Research has shown that a higher mold temperature can reduce the occurrence of defects such as sink marks and improve the overall surface quality of the molded part [21,23].

The design and location of the gate are equally significant in determining the flow characteristics of the injected material. A well-positioned gate can facilitate a more uniform filling of the mold cavity, reducing the likelihood of defects such as weld lines and sink marks. Research indicates that the gate position can influence the flow path and the resultant pressure distribution within the mold, which is critical for achieving dimensional accuracy [24,25,26]. For example, improper gate placement can lead to uneven cooling rates and shrinkage, resulting in warpage and other dimensional defects [26,27]. Moreover, the distance from the gate can create variations in cooling rates, which are essential for the crystallization behavior of certain polymers, further affecting the mechanical properties of the molded parts [28].

Studies have demonstrated that optimizing holding pressure can significantly enhance the consistency and quality of molded parts by ensuring that the cavity remains filled during the cooling phase [22,27,29]. The relationship between holding pressure and the distance from the gate is particularly noteworthy; parts closer to the gate may require different holding conditions compared to those further away due to differences in thermal history and material flow characteristics [30].

In conclusion, the influence of injection molding parameters and the distance from the gate is a multifaceted issue that encompasses various aspects of material science and engineering. The interplay between injection speed, pressure, cooling rates, and gate design significantly affects the mechanical properties and dimensional accuracy of the final product. Ongoing research in this field continues to uncover new insights and optimization strategies, highlighting the importance of a comprehensive understanding of these factors for achieving high-quality injection-molded products.

The literary research showed that no currently existing work is concerned with the effect of melt flow length and injection parameters on the mechanical properties of the part. In most cases, the mechanical properties and eventually the hardness are determined in the single location, and the result of these measured properties is accepted as the final property in the whole volume of the injected part. However, no similar study has been found on the issue of different properties along the injection-molded products. In the studies mentioned above, only partial observations are described, which deal with the influence of the injection molding process parameters on the mechanical properties, or the influence of the change in crystallinity, but, again, usually at one location of the tested part. The interplay between injection speed, pressure, cooling rates, and gate design significantly affects the mechanical properties and dimensional accuracy of the final product. This study focuses on the effect of melt flow length from the gate locations and process parameters on the selected mechanical properties of the injection-molded part.

## 2. Materials and Methods

### 2.1. Overview of the Experiment (DOE)

The parameters of DOE were limited by a number of factors, resulting in the use of central composite design with 14 randomized runs, which were set for the range of the used equipment. The choice of a central composite design with 14 randomized runs was made based on the need to efficiently model quadratic effects for the two key factors (temperature and pressure) while maintaining statistical robustness. The number of runs (14) was limited by the structure of a two-factor CCD, which requires a combination of factorial, axial, and center points. The parameter ranges (temperature, pressure) were constrained by equipment capabilities as well as safe operating conditions.

The same methodology that was used to plan and measure the effects of pressure and distance on resulting H_IT_ was also used to measure the change within observed mechanical properties for different mold temperatures.

### 2.2. Material

The tested material was a semi-crystalline thermoplastic polypropylene material with the trade name BJ380MO, which was supplemented by Borealis (Linz, Austria). The selected basic parameters are given in Table 1; the parameters are from the polypropylene material sheet.

### 2.3. Injection Mold

**Figure 1:**
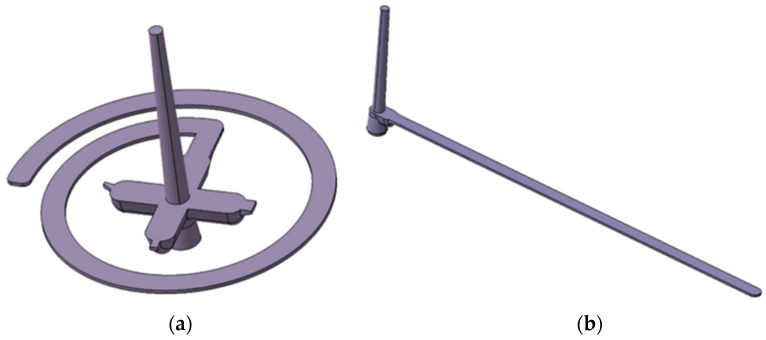
Test samples: (**a**) spiral, (**b**) developed spiral.

The test samples were produced on 2 injection molds. The injection mold cavity was a spiral shape (Figure 1a) with a length up to 2000 mm (designed so that the melt does not reach the end of the spiral, for comparison purposes). The spiral-shape cavity mold was used to determine the flowability of polymers. The length of the spiral greatly exceeded the polymer filling capabilities at the specified process parameters. Due to the fact that the spiral cavity was not fully filled with the polymer and could lead to inaccurate results, test mold 2 was designed to ensure full filling of the cavity. The second mold was designed to verify the results from the spiral mold (Figure 1b). It was a mold cavity in the shape of a developed spiral in 4 length variations defined by the standard, where filling of the mold cavity was ensured, and the mold cavity had the same cross-section as the spiral cavity. The single cavities were designed to allow the polymer to fill the entire mold cavity with different injection molding process parameters set. The 2 mold cavities had lengths of 200 mm, 150 mm, 60 mm, and 30 mm. The measurement results on both test specimens showed the same values. For this reason, the results from the spiral injection mold (mold 1) were presented in this paper. The cross-section of the mold cavity was 6 × 1 mm. The test samples are shown in Figure 1.

### 2.4. Sample Preparation

The injection molding machine used to produce the test samples was the Allrounder 470E 1000-290 Golden Edition from Arburg (Losburg, Germany). The Regloplas 150 smart oil tempering unit manufactured by Regloplas (St. Gallen, Switzerland) was used to temper the molds. By using the recommended values from the polypropylene material sheet in accordance with the injection molding machine capabilities and based on the values obtained from the injection molding process simulations, the process parameters shown in Table 2 were selected. The selected parameters were varied in Table 3.

### 2.5. Mechanical Properties

**Figure 2:**
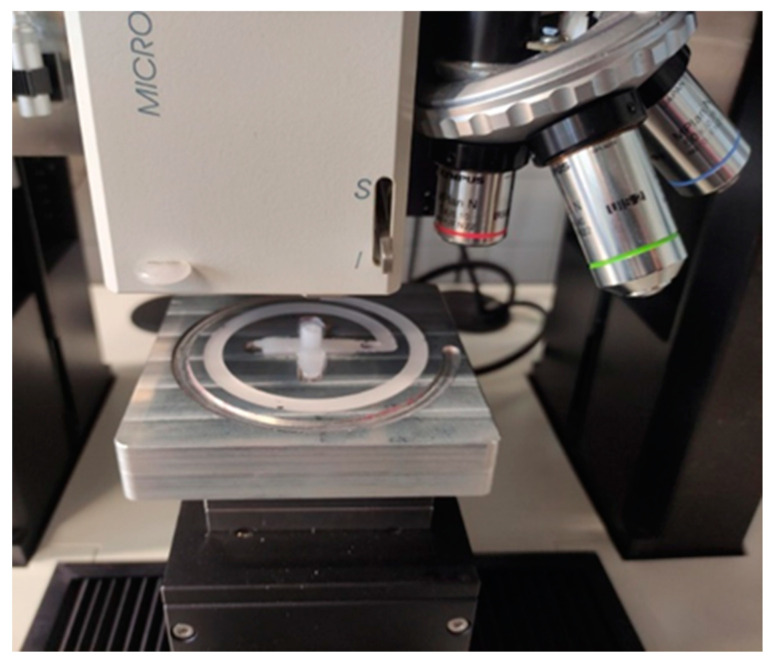
Measurement of micro-mechanical properties.

The mechanical properties were measured at a maximum of 6 distances (0 mm, 78 mm, 157 mm, 196 mm, 225 mm, 267 mm) when the melt reached this distance under the specified conditions. The individual distances were chosen with respect to the length of the run-in at different injection pressures. For an injection pressure of 20 MPa, the length of penetration was 157 mm. Based on this, 3 measurement areas were selected, namely right at the inlet (0 mm), at the end of the inflow (157 mm), and in the middle (78 mm). For an injection pressure of 40 MPa, the length of inflow was 196 mm. The measurement locations from the previous pressure were retained, and a location at the end of the inflow of 196 mm was added (4 measurement areas). For an injection pressure of 60 MPa, the length of inflow was 225 mm. The measurement locations were the same as for the 40 MPa injection pressure, and the final run-in length of 225 mm was added (5 measurement areas). For 80 MPa injection pressure, 6 measurement areas were set, which were the same as for 60 MPa injection pressure, and a final measurement at the end of the inflow of 267 mm was added. The reason for this specification of measurement areas was to capture the change in mechanical and structural properties along the length of the test specimens. And also, to be able to compare properties at different injection pressures in the same measurement areas. Mechanical properties were measured using an instrumented hardness test on a Micro-combi Tester (Figure 2) from Anton Paar (Graz, Austria). Table 4 shows the set measurement parameters. A Vickers indenter in the shape of a quadrilateral diamond pyramid with a peak angle of 136° was used as a hardness indicator. The measurements were performed by Depth Sensing Indentation (DSI), and the evaluation of the mechanical properties was performed by the Oliver and Pharr method. The measurements were done according to standard ČSN EN ISO 14577 [31,32,33,34]. For the micro-hardness test, low loading forces were applied with a measurement depth of the order of µm; only the indentation hardness, indentation modulus, and indentation creep were evaluated from the measured mechanical properties.

As delineated in ISO 14577 [31,32,33,34], the following parameters were the focus of the evaluation: indentation hardness, modulus, and creep. The calculation of the individual values was conducted employing the Oliver and Pharr method. Indentation hardness *H_IT_* can be defined as the ability of a material to resist plastic deformation. The expression for indentation hardness *H_IT_* (Equation (1)) is derived by dividing the applied load, *F_max_*, by the contact area, *A_p_*, between the indenter and the specimen at maximum depth and load. The contact area, *A_p_*, is determined by the constant of the indenter shape (24.50 for the Vickers indenter) and the indenter’s depth of contact with the specimen, *h_c_* [31,32,33,34].

(1) $$H_{IT}=\frac{F_{max}}{A_{p}}$$

(2) $$A_{p}=24.50\cdot h_{c}^{2}$$

The DSI method of indentation testing facilitates the acquisition of additional material properties, including the indentation modulus *E_IT_* (Equation (3)). The indentation modulus *E_IT_* is an elasticity value and the most important parameter for all applications with elastic materials. The modulus *E_IT_* is calculated from the unloading curve of the indentation. In many cases, *E_IT_* values are comparable to the classic modulus of elasticity. As outlined in the ISO 14577 [31,32,33,34] standard, the reduced modulus, *E_r_*, is employed to account for the phenomenon that elastic displacements occur in both the indenter and the specimen. This is achieved by applying the projection area *A_p_* to the calculation of the Reduced Modulus *E_r_* (Equation (5)), which eliminates the elastic behavior of the tested material. The instrumented elastic modulus in the test material, *E_IT_*, can be calculated from *E_r_* (Equation (5)). The calculations involve Poisson’s ratio (*v_s_*), which is typically between 0.2 and 0.4 for metallic materials and 0.3 to 0.4 for polymeric materials [31,32,33,34].

The plane strain modulus, E*, is calculated using the following Equation (4), where E_i_ is the elastic modulus of the indenter (1141 GPa for diamond), *E_r_* is the reduced modulus of the indentation contact, and v is Poisson’s ratio of the indenter (0.07) [31,32,33,34].

Reduced modulus *E_r_* is calculated from the following Equation (5), where C is contact pliability and *A_p_* is contact area $\sqrt{A_{p}}=4.950\;*\;h_{c}$ for the Vickers indenter [31,32,33,34].

(3) $$E_{IT}=E^{*}\cdot (1-v_{s}^{2})$$

(4) $$E^{*}=\frac{1}{\frac{1}{E_{r}}-\frac{1-v_{i}^{2}}{E_{i}}}$$

(5) $$E_{r}=\frac{\sqrt{\pi }}{2\cdot C\sqrt{A_{p}}}$$

Indentation creep is defined as the relative change in indentation depth whilst the applied load remains constant. This concept is formally defined in the ISO 14577 [31,32,33,34] instrumented indentation standard as *C_IT_* (see Equation (6)). In this equation, h_1_ denotes the depth at the commencement of the creep test and h_2_ denotes the depth at the conclusion of the creep test [31,32,33,34].

(6) $$C_{IT}=\frac{h_{2}-h_{1}}{h_{1}}100$$

All the measurements of mechanical properties were carried out 9 times and then they were statistically evaluated.

### 2.6. Differential Scanning Calorimetry (DSC)

The differential scanning calorimeter DSC Q20 (TA Instruments, New Castle, DE, USA) the behavior of the test bodies was investigated at melting and solidification. The weight of the bulking material was 6 mg, and the required amount was separated using microtome cuts. The heating and cooling rate was set at 10 °C/min. The measurement was divided into two parts. The 1st part of the measurements consisted of a 1st heating from T_0_ to T_1_, followed by a time delay at constant temperature (isotherm 1 min), then cooling to T_0_, another time delay at constant temperature (isotherm 1 min), and then followed by heating to T_1_. The properties monitored were determined during the 1st heating.

The crystallinity was calculated from the heat fluxes according to the following equation [35]:

(7) $$X_{c}=\frac{∆H_{m}}{{∆H}_{m}^{100}}\times 100$$

where *X_c_* is crystallinity (%), Δ*H*_m_ is heat flux (J/g), and Δ*H*_m_^100^ is heat flux for 100% crystalline polypropylene (207 J/g), found in the literature [35].

Differential scanning calorimetry DSC was measured 5 times for each variant and then statistically evaluated.

## 3. Results

The specimens were manufactured by injection molding technology. During the manufacturing process, the technological parameters and mold plates were varied. For each setting, 9 samples were created and the mechanical properties (indentation hardness, indentation modulus, and indentation creep) were measured. The mechanical properties were obtained from the results of an instrumental hardness test using the Oliver and Pharr method. The measured data were then statistically evaluated. Since all the plate variants had similar tendencies, the Ra 0.8 µm ground plate was chosen as an example.

### 3.1. Overview of the Experiment

**Figure 3:**
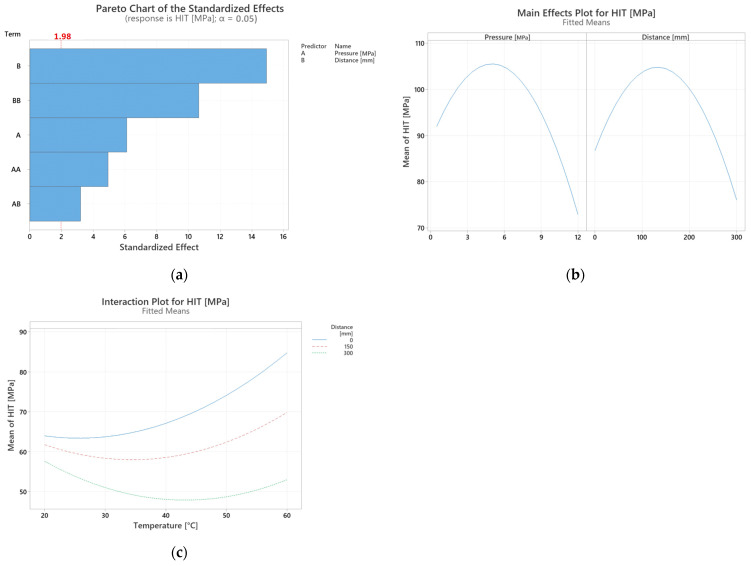
(**a**) Pareto chart of the standardized effects (response is indentation hardness H_IT_, α = 0.05), (**b**) Main effects plot for indentation hardness H_IT_ (fitted means), (**c**) Interaction plot for indentation hardness H_IT_.

The Pareto Chart of the Standardized Effects confirms that both main parameters are statistically significant for the observed indentation hardness (*H_IT_*) values. The largest effect is caused by the “distance”, while the pressure itself isn’t as crucial as can be seen in Figure 3a. What is of interest is the fact that the combination of “distance” and “pressure” has the least significant effect on the resulting hardness.

Figure 3b is the result of the two-level full factorial Design of Experiment method. The figure itself describes the dependence of indentation hardness H_IT_ on pressure and distance, respectively. As is clear from the results of the DOE, the mean H_IT_ is severely impacted at very high pressures, which also negatively affects the performance of test samples at larger distances. The optimal range for pressure settings was thus reduced to 80 MPa. At very low pressures, such as 50 MPa, the achievable distance the test samples could be developed to was also very low, although with better mechanical properties than at very high pressures. In order to reliably test at least three distances for each pressure, the lower limit for tested pressure was set to 20 MPa. The resulting range of testing was based on the above and set to 20–80 MPa. At 80 MPa the longest reliable distance for the test sample was 267 mm. In order to save time and resources, the tests were conducted with a 20 MPa gap as the difference between; for e.g., 20 and 30 MPa was not significant enough to warrant such fine measurements.

Although it was possible to reach flow distances up to 267 mm for each mold temperature, the mean H_IT_ for distances higher than 208 mm were not of much interest due to their poor performance. Based on this observation, the following distances were selected for further testing. Figure 3c) states the interactions between temperature and distance on the mean H_IT_. As is clear from the graph, the distance has a very strong effect on the mean H_IT_, especially in combination with higher mold temperatures where the variation between mean H_IT_ is most significant. This trend is less pronounced in the lower mold temperature region. Further testing was then needed to ascertain the full scope of effects of both distance and temperature.

### 3.2. Influence of Injection Pressure and Flow Length on Mechanical Properties

When investigating the effect of injection pressure, the pressure was modified according to the recommendations from the polypropylene material sheet. Mechanical properties when investigating the effect of injection pressure were measured at the following distances: 0 mm, 78 mm, 157 mm, 196 mm, 255 mm, and 267 mm. These distances were chosen with respect to the length of the flow at different injection pressures (for injection pressure 20 MPa, the length of the flow was 157 mm; for injection pressure 40 MPa, the length of the flow was 196 mm; for injection pressure 60 MPa, the length of the flow was 255 mm; and for injection pressure 80 MPa, the length of the flow was 267 mm). The reason for these measurement locations was to capture the changes in mechanical and structural properties along the entire length of the test specimens. And, also, to be able to compare properties in the same locations of the test specimens at different injection pressures.

**Figure 4:**
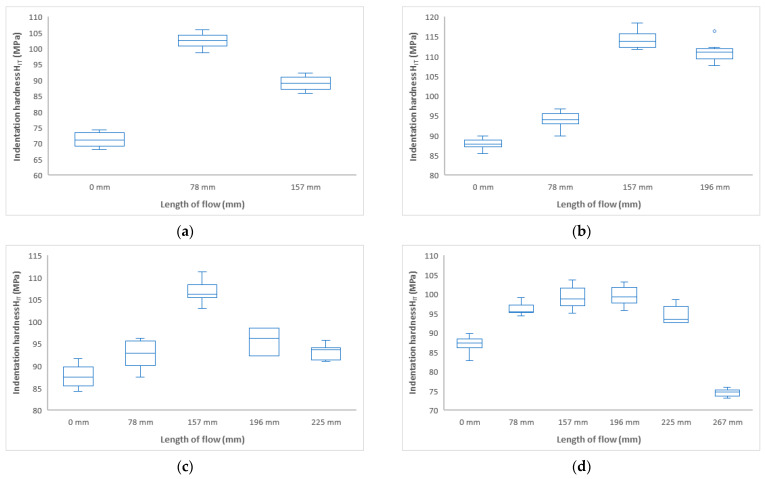
Dependence of indentation hardness H_IT_ on flow length at injection pressures (**a**) 20 MPa, (**b**) 40 MPa, (**c**) 60 MPa, and (**d**) 80 MPa.

An important property describing the influence of the melt flow length on the properties of the manufactured part is the indentation hardness H_IT_ (MPa). Figure 4 shows that the indentation hardness varies along the length of the test sample for all set variations. For all injected samples, an increase in indentation hardness was observed from the 0 mm inlet gate to the middle of the test sample with a subsequent decrease.

At the lowest selected injection pressure of 20 MPa, the injection hardness (Figure 4a) at the inlet (0 mm distance) was found to be approximately 72 MPa. Towards the center of the sample (distance 78 mm), the indentation hardness increased significantly to 103 MPa. Near the end of the part, the indentation hardness was measured to drop to 89 MPa. The indentation hardness increased along the flow path by 43% (distance 78 mm) and by 24% (distance 157 mm).

Similar trends were found for an injection pressure of 40 MPa (Figure 4b). The inlet location (0 mm) showed the lowest hardness values of 88 MPa and towards the middle of the part the values of the indentation hardness increased up to 157 mm, where a hardness of 114 MPa was measured. At the end of the part, a slight decrease in indentation hardness values—to 111 MPa—was observed. The difference between the inlet location and the end of the part was 26%.

At injection pressures of 60 MPa and 80 MPa, similar indentation hardness curves were measured along the length of the product (Figure 4c,d). The injection inlet exhibited indentation hardness values of approximately 87 MPa. The maximum indentation hardness was measured at 157 mm and ranged from 100 to 106 MPa. The increase in indentation hardness was 22%. Towards the end of the part, a significant decrease in indentation hardness was observed, approaching the value measured at the point near the sprue gate.

The observed changes in the mechanical properties are due to the distribution of the crystalline phase (Section 3.2.1) and the change in the arrangement of the structure (size and shape of the spherulites) along the product flow, where the size of the hold pressure and the temperature of the mold have major influence. Both these parameters have been studied, and their influence on the heterogeneous behavior of the mechanical properties has been demonstrated. In technical practice, the product is usually taken as a whole with uniform properties, but this does not always correspond to reality, as the properties may vary at different locations. With a suitable choice of gate location and process parameters such as hold pressure or mold temperature, it is possible to achieve better properties at a predetermined location than at other part locations. This can be applied to stressed local areas of the final product; these areas can be specifically modified without the need for a better (more expensive) material.

These findings are consistent with the measured crystalline phase change presented below in Section 3.2.1.

**Figure 5:**
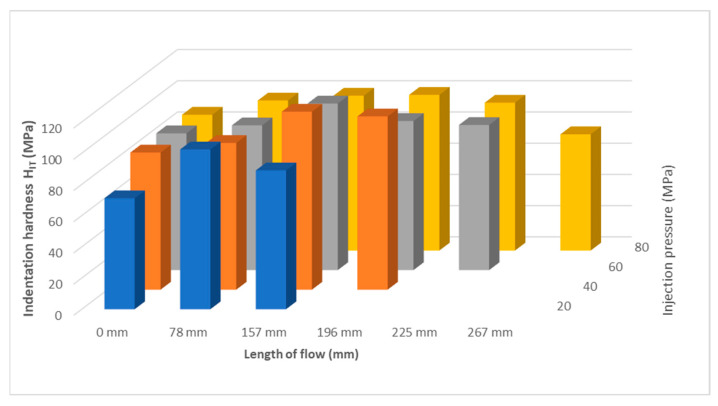
Dependence of indentation hardness H_IT_ on flow length and injection pressure.

Comparing the results of the indentation hardness at different injection pressures, it can be seen that the pressure has an effect on the mechanical properties along the length of the sample, in addition to the effect on the melt flow length. As can be seen in Figure 5, with increasing pressure, the length of the run-in increased, as did the length where the highest value of the indentation hardness occurred. The magnitude and course of the injection pressure have an effect on the formation of the final product structure, which determines the shape and properties.

**Figure 6:**
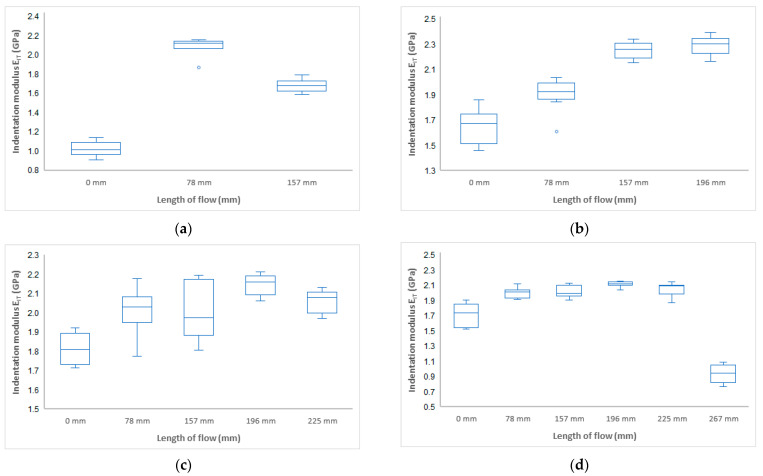
Dependence of indentation modulus E_IT_ on flow length at injection pressure (**a**) 20 MPa, (**b**) 40 MPa, (**c**) 60 MPa, and (**d**) 80 MPa.

**Figure 7:**
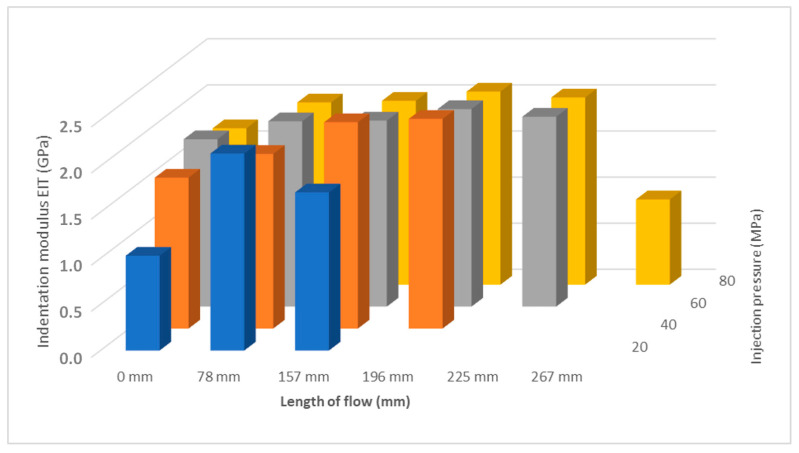
Dependence of indentation modulus E_IT_ on flow length and injection pressure.

Another important mechanical property determined from the instrumental hardness test is the indentation modulus E_IT_ (GPa). The indentation modulus ideally corresponds to the Young’s modulus of elasticity. The results of the measurement of the indentation modulus for samples injected with 20 MPa, 40 MPa, 60 MPa, and 80 MPa can be seen in Figure 5. It can be seen that the results of the indentation modulus (Figure 6) correspond with the results of the indentation hardness measurements (Figure 4). Results show that the highest modulus is in approximately half of the samples for all pressure settings (Figure 7).

For an injection pressure of 20 MPa, the minimum value of the indentation modulus of 1 GPa was measured at the inlet (distance 0 mm), and, towards the middle of the part (distance 78 mm), an increase to 2.2 GPa was measured. The difference between the distance of 0 mm and 178 mm was 120%. At the end of the part, a decrease in the indentation modulus was measured. At an injection pressure of 40 MPa, the indentation modulus near the inlet was measured to be 1.7 GPa, and towards the end of the part, an increase to 2.3 GPa was measured, which is 35%. For injection pressures of 60 MPa and 80 MPa, values of about 1.8 GPa were measured near the inlet and towards the end of the part; up to a distance of 196 mm, a maximum was found with a value of about 2.1 GPa. This difference was 16%. At the end of the section, a significant decrease was measured.

**Figure 8:**
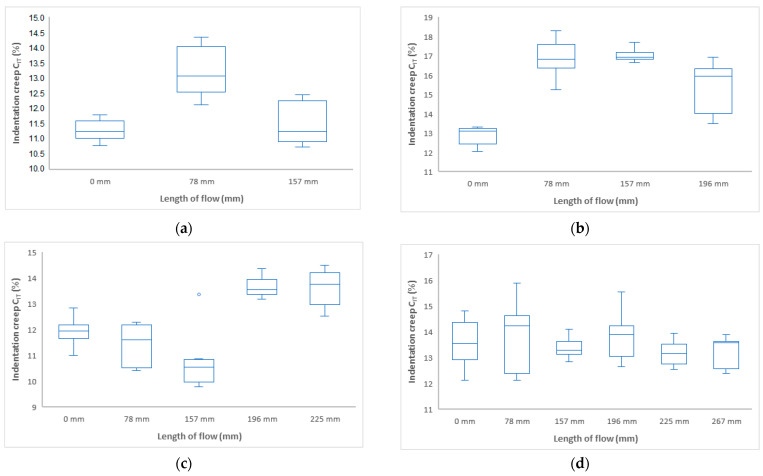
Dependence of indentation creep C_IT_ on flow length at injection pressure (**a**) 20 MPa, (**b**) 40 MPa, (**c**) 60 MPa, and (**d**) 80 MPa.

**Figure 9:**
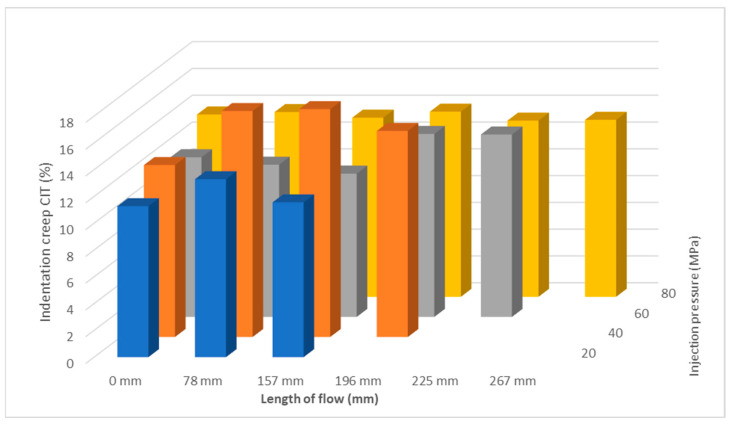
Dependence of indentation creep C_IT_ on flow length and injection pressure.

Indentation creep C_IT_ (%) has the same tendency as the other two measurements in terms of mechanical properties (Figure 8 and Figure 9). The highest value of indentation creep was found for the sample injected at 40 MPa at 157 mm 17%.

From the above results, it is evident that the injection pressure affects the mechanical properties at different melt flow lengths. Increasing the injection pressure has a positive effect on the melt flow length of the polymer, which also affects the change in mechanical properties at different flow lengths. In most of the measurement cases, the maximum property value found was approximately in the middle of the flow distance. The value of the injection pressure and its course has a significant effect on the final product. It is involved not only in filling the mold cavity, but also in the formation of the final structure of the injected parts, which will affect the shape and properties of the product.

**Figure 10:**
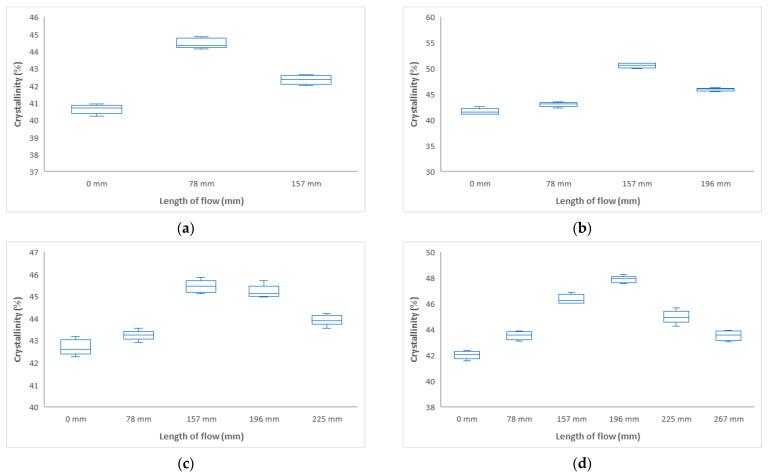
Dependence of crystallinity on flow length at injection pressure (**a**) 20 MPa, (**b**) 40 MPa, (**c**) 60 MPa, and (**d**) 80 MPa.

From the present results, it can be considered that the change in injection pressure has a positive effect on the length of flow, but it is also reflected in the change in mechanical properties at different distances from the inlet (Figure 10). Due to the shorter flow path, and thus a more uniform temperature field in the mold cavity at lower injection pressures, higher values of mechanical properties were measured. Changes in mechanical properties were also observed at different distances from the inlet, where the highest increase in mechanical property values (indentation hardness and indentation modulus) was measured at approximately the center of the test body. At lower pressures (20 and 40 MPa), no significant decrease in properties was observed, while at higher pressures, the decrease was significant. These changes can be attributed to the different heat-temperature fields during filling and cooling in the mold.

#### 3.2.1. Influence of Injection Pressure and Flow Length on Crystallinity (DSC)

From the results of the DSC measurements at different distances from the inlet, it is clear that the volume of the crystalline phase, and hence the mechanical properties, change along the flow, as shown in Figure 10. This change corresponds exactly to the change in mechanical properties. As the polymer is cooling in the mold cavity at different distances from the inlet, different crystallization occurs, where, in addition to the arrangement of the molecules, spherulites are formed at different locations with different properties.

From the above results of crystallinity determined by DSC, it can be concluded that the crystallinity varies at different distances from the inlet, as shown in Figure 10. These findings correspond with the change in mechanical properties. For all injection pressures, the crystallinity was measured to be lowest at the inlet, ranging from 41 to 42%. Towards the center of the sample, an increase was measured up to 52% (distance 157 mm at 40 MPa), 46% (distance 157 mm at 60 MPa), and 48% (distance 196 mm at 80 MPa). The change in crystallinity is due to the polymer flow through the mold cavity, cooling of the polymer in the mold cavity, and the injection molding process parameters.

### 3.3. Effect of Mold Temperature and Flow Length on Mechanical Properties

In this part, the influence of the distance from the sprue at different mold temperatures (30 °C, 40 °C, 50 °C, 60 °C) on the mechanical properties was investigated. When investigating the effect of mold temperature, the temperature was varied according to the recommendations of the polypropylene material data sheet. Mechanical properties when investigating the effect of mold temperature were measured at the following distances: 0 mm, 76 mm, 154 mm, 192 mm, and 208 mm.

The finding in Section 3.1 showed that the mechanical properties (indentation hard-ness, indentation modulus, and indentation creep) are closely related to the crystalline phase formed, the formation of which is affected by the heat of cooling in the mold cavity. Therefore, the effect of flow length on the mechanical properties and crystallinity at different mold temperatures was investigated.

**Figure 11:**
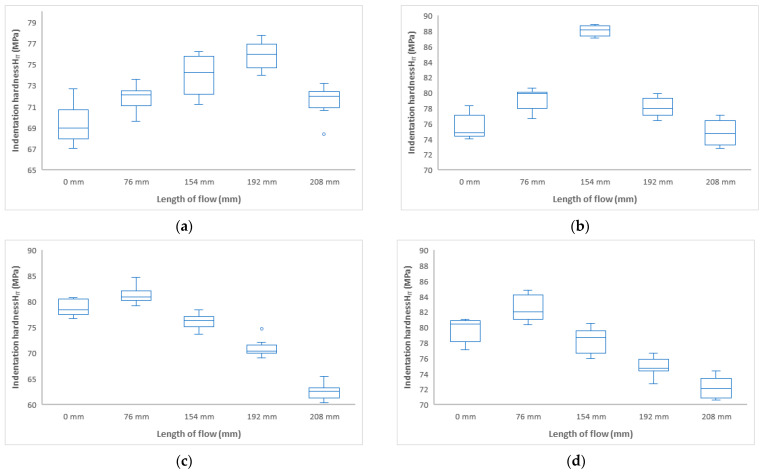
Dependence of indentation hardness H_IT_ on flow length at mold temperature: (**a**) 30 °C, (**b**) 40 °C, (**c**) 50 °C, (**d**) 60 °C.

From the result shown in Figure 11a,b, it can be seen that at mold temperatures of 30 °C and 40 °C, the indentation hardness first increased to approximately the middle of the distance of the injected sample (at 192 mm distance, hardness 76 MPa, and at 154 mm distance, hardness 88 MPa), and then a decrease in indentation hardness was observed towards the end of the part, to values close to the values at the inlet (69 to 75 MPa). The difference between the sprue, the end of the part, and the center of the part was 17%.

At mold temperatures of 50 °C and 60 °C, a decrease in indentation hardness towards the end of the part was observed, as shown in Figure 11c,d. The decrease in hardness compared to the injection point (78 to 81 MPa) and the end of the part (62 to 72 MPa) was approximately 24%.

Comparing the indentation hardness at the point of the injection gate at 30 °C (69 MPa), 40 °C (75 MPa), 50 °C (78 MPa), and 60 °C (81 MPa), it is evident that higher mold temperature results in an increase in indentation hardness. Towards the end of the parts, temperatures of 30 and 40 °C cause an increase in the indentation hardness and a subsequent decrease. At temperatures of 50 and 60 °C, the indentation hardness decreases along the entire length of the product. The optimum temperature appears to be 40 °C, where the values are highest and constant over the entire length.

**Figure 12:**
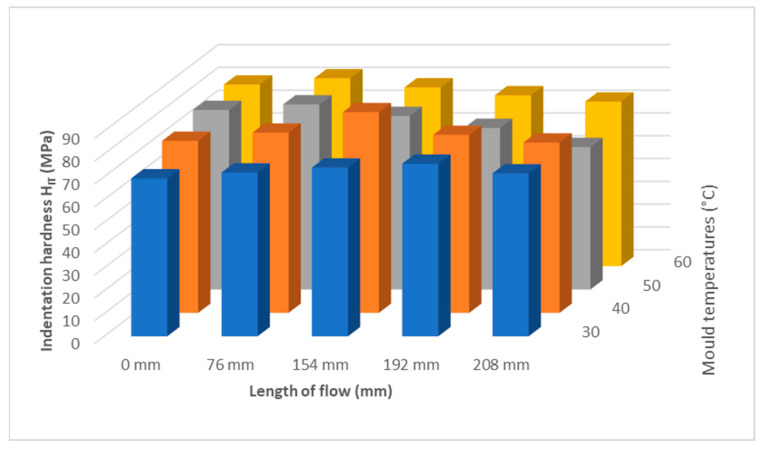
Dependence of indentation hardness H_IT_ on flow length and mold temperature.

Figure 12 shows that the mold temperature has an effect on the indentation hardness. It is also noticeable that at lower mold temperatures of 30 °C and 40 °C the hardness nearer the gate is lower than at higher mold temperatures of 50 °C and 60 °C. This trend is reversed approximately as the sample length increases, and the indentation hardness is higher at lower mold temperatures of 30 °C and 40 °C.

**Figure 13:**
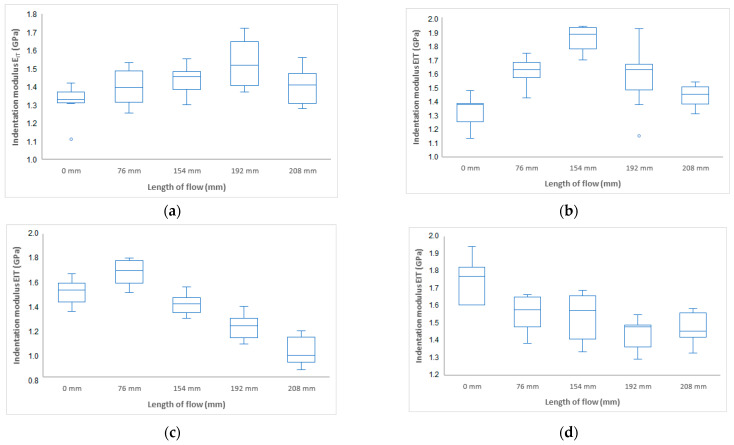
Dependence of indentation modulus E_IT_ on flow length at mold temperature: (**a**) 30 °C, (**b**) 40 °C, (**c**) 50 °C, (**d**) 60 °C.

**Figure 14:**
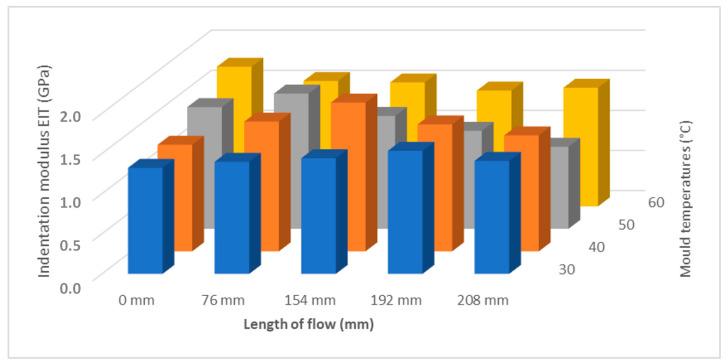
Dependence of indentation modulus E_IT_ on flow length and mold temperature.

The results of the indentation module on the distance from the sprue mouth at different mold temperatures are shown in Figure 13 and Figure 14. The results have a similar trend to the indentation hardness. The highest value was measured on the sample at a temperature of 40 °C at a distance of 154 mm.

**Figure 15:**
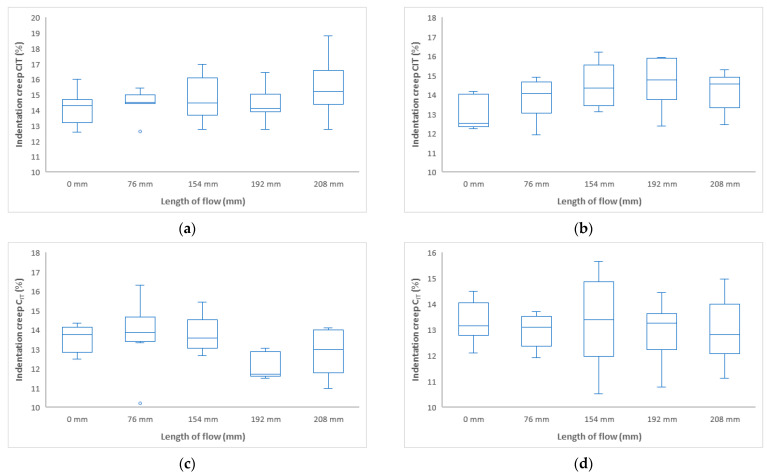
Dependence of indentation creep C_IT_ on flow length at mold temperature (**a**) 30 °C, (**b**,**c**) 40 °C, 50 °C, and (**d**) 60 °C.

**Figure 16:**
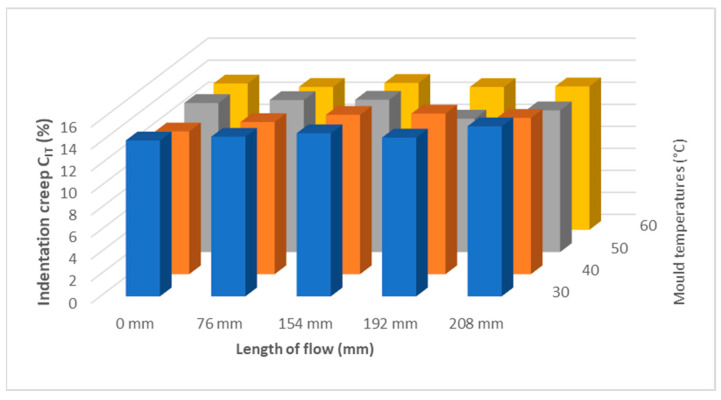
Dependence of indentation creep C_IT_ on flow length and mold temperature.

Figure 15 and Figure 16 shows indentation creep results as a function of flow length at different mold temperatures. The results for indentation creep show that they do not vary too much along the length of the sample.

As can be seen in Figure 11, the mold temperature has an effect on the mechanical properties of the samples. Lower mold temperatures of 30 °C show lower mechanical properties and do not appear to be suitable. From a temperature of 40 °C onwards, the properties stabilize and no significant differences in mechanical properties can be observed.

Considering the results of the influence of the distance/flow length from the inlet at different mold temperatures, it is evident that the mechanical properties are influenced by the mold temperature. At a temperature of 30 °C, the maximum was measured at a distance of 192 mm. The difference between the sprue gate and this distance is 11%. At a temperature of 40 °C, the maximum in mechanical properties was measured at a distance of 154 mm. The increase in the mechanical properties was up to 18%, and, at greater distances from the inlet, the decrease was to the same values as at the inlet. At mold temperatures of 50 °C and 60 °C, only a slight increase in mechanical properties was measured at a distance of 76 mm, followed by a decrease of up to 24% up to the end of the part.

From the results of the mold temperature measurements, it can be seen that a temperature of 40 °C appears to be suitable for injection molding of polypropylene. No significant decrease along the length of the product was measured; rather, there was an improvement in the mechanical properties in the middle of the part. These results are consistent with the change in crystallinity described in the following section.

#### Influence of Mold Temperature and Flow Length on Crystallinity (DSC)

**Figure 17:**
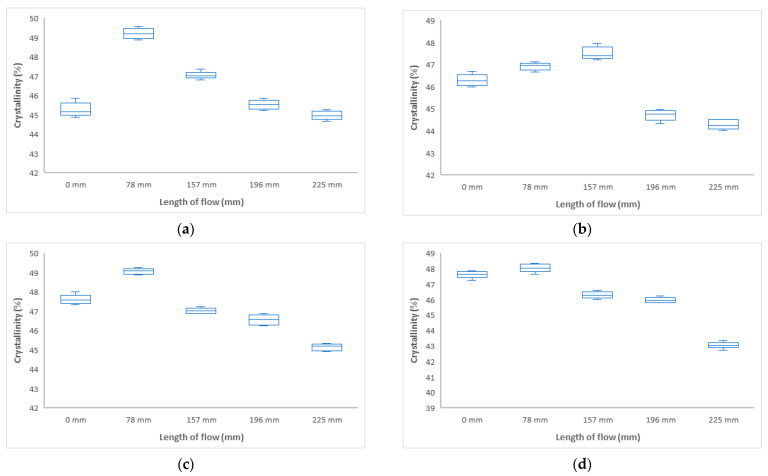
Dependence of crystallinity on flow length at mold temperature: (**a**) 30 °C, (**b**) 40 °C, (**c**) 50 °C, (**d**) 60 °C.

In this section, the effect of the distance from the inlet on the change in the volume of the crystalline phase, which was measured by DSC, was investigated. Figure 17 shows the DSC measurements and the volume change of the crystalline phase at different distances from the inlet at mold temperatures of 30 °C, 40 °C, 50 °C, and 60 °C.

Polymer flow, mold temperature, and melt face temperature are important parameters involved in the formation of the final polypropylene structure. These parameters have a significant effect on the heterogeneity of properties at different points in the product. By setting these parameters, it is possible to tailor the formation of the structure at different points of the product and thus influence the mechanical properties of the final product. From the above results, it can be seen that the volume of the crystalline phase increases with increasing mold temperature (from 30 °C to 60 °C). There is a significant increase up to 50 °C, and, from this temperature onwards, the crystallinity values stabilize (Figure 17). Changes in crystallinity are also evident at different distances from the inlet. These results correspond to the change in mechanical properties (indentation hardness and indentation modulus).

From the results of DSC measurements at different distances from the inlet, it is clear that the volume of the crystalline phase, and hence the mechanical properties, change along the flow, as shown in Figure 17. This change corresponds exactly to the change in mechanical properties. As the polymer cools in the mold cavity at different distances from the inflow, different crystallization occurs, with different sizes of spherulite nuclei forming with different properties in different mixtures, depending on the arrangement of the molecules

Research indicates that the molecular orientation of polymers tends to be more pronounced near the gate due to the high shear rates experienced during injection. This phenomenon results in anisotropic properties, where the mechanical characteristics differ based on the orientation of the material [36]. Studies have shown that parts closer to the gate tend to cool more rapidly, leading to different crystallinity levels compared to those farther away [26,28]. For instance, Björn et al. demonstrated that polymers exhibit a higher degree of orientation close to the injection gate, which correlates with increased mechanical strength in that region [36].

A schematic diagram of the mechanism of the development of hierarchical structures in the surface (skin) layer and deeper in the (core) layer during mold cavity filling and subsequent cooling in the mold was shown by the authors in [37]. In the injection molding process, the important phases are mainly the polymer flow and the polymer temperature (cooling), where the orientation of the molecular chains in the direction of the flow during mold filling occurs as the melt cools. In the core layer, the longer chains can remain in the stretched state, while the short chains are randomly oriented during the mold-filling phase.

## 4. Discussion

The results presented in this paper describe the influence of the distance from the gate and process parameters on the mechanical properties of an injection-molded polypropylene part. The properties were measured at pre-specified locations along the length of the melt flow (0 mm, 78 mm, 157 mm, 196 mm, 255 mm, and 267 mm if the melt reached these distances under the specified conditions) and describe the behavior of the polymer melt in the mold cavity. The research is based on an industrial part for which different mechanical properties were measured at different locations. Based on the above, the testing procedure was established with the proposed measurement methods. The test bodies were prepared in a spiral mold. The boundaries of the research consisted of the selected material, polypropylene BJ380MO (Borealis), according to which the selected injection molding parameters were obtained from the recommendations in the material data sheet of polypropylene.

The polymer flow in the injection mold cavity is affected by process parameters besides surface quality, which include injection pressure and mold temperature. Both process parameters were varied according to the material manufacturer’s recommendations and also according to the capabilities of the injection molding machine and tool.

The measurement results were statistically evaluated using selected statistical parameters and displayed in boxplot diagrams. The measurement of the influence of injection pressure (20 MPa, 40 MPa, 60 MPa, and 80 MPa) on mechanical properties shows significant similarity in trend to mechanical properties at varying distances from the gate along the length of the injected sample. The indentation hardness H_IT_ increased with the length of the injected sample up to the middle of its length, followed by a decrease in hardness for all set values of injection pressure. This increase in mechanical properties was up to 37% in some cases. By contrast, a significant decrease in mechanical properties was observed towards the end of the test body. The results of indentation modulus E_IT_ and indentation creep C_IT_ have the same tendency as the results of indentation hardness measurements. The highest values of mechanical properties were achieved for samples injected at an injection pressure of 40 MPa.

For measuring the effect of temperature (30 °C, 40 °C, 50 °C, and 60 °C) on the mechanical properties of the injection-molded sample along its length, the temperature was varied according to the recommendations in the polypropylene data sheet. The trend of indentation hardness H_IT_ and indentation modulus E_IT_ are similar, at first increased up to approximately the middle of the distance of the injected sample and then decreased to the end of the sample. For indentation hardness H_IT_, it is also noticeable that at lower mold temperatures (30 °C, 40 °C), the hardness nearest the gate is lower than at higher mold temperatures (50 °C, 60 °C). This trend is approximately reversed as the sample length increases, and the indentation hardness is higher at lower mold temperatures. The results show that indentation creep C_IT_ does not vary much along the length of the injected sample. The highest values of mechanical properties were achieved for samples injected at mold temperatures of 40 °C. Comparing a temperature of 30 °C with 40 °C and above, the mechanical properties increased by up to 17%.

As the results show, the melt flow distance has a significant effect on the mechanical properties. These properties vary along the length of the product and are due to the change in crystallinity at different distances from the flow path. The results were influenced by the melt flow length but mainly by the injection molding process parameters. These parameters affected the conditions of filling the mold cavity and the formation of the resulting skin–core structure, which is variable along the length of the product and contributes to the heterogeneity of the mechanical properties. The resulting skin–core structure, which is influenced by the degree of orientation and crystalline morphology in the different layers of the part, has a major influence on the observed properties at different points of the product. These changes can be influenced by changing the injection pressure and especially the temperature profile in the mold cavity. From the results, it can be concluded that the changes in mechanical properties, which are influenced by the resulting morphology of polypropylene, can be captured using the indentation method. This morphology can be correlated with the mechanical properties. It is also important to pay attention to the setting of process parameters, especially the mold temperature, which has a major influence on the heterogeneity of the mechanical properties.

The contribution of this work is the investigation of the mechanical properties of an injection-molded polypropylene part as a function of the distance from the gate location and the injection process parameters (injection pressure and mold temperature). The work is suitable for application in engineering practice because it does not consider the homogeneity of properties over the whole injected part but provides insights into the inhomogeneity of properties of injection-molded parts. Based on the findings of this work, the final properties of the injection-molded part can be modified by structural modification of the mold or by changing the injection process parameters. The appropriate choice of the location of the gate can affect the mechanical properties along the part, but the temperature of the mold also has an effect on these properties. Hence, by changing the location of the sprue mouth and possibly changing the trajectory of the tempering circuit, we can significantly affect the mechanical properties in a specific area of the injection-molded part.

## 5. Conclusions

This paper deals with the study of the effect of melt injection distance, tool surface, and process on the properties of a part made by polypropylene injection molding technology. In this paper, the injection molding process parameters such as injection pressure and mold temperature were varied. The mechanical properties (indentation hardness and indentation modulus) and crystallinity (DSC method) were studied on the test bodies prepared in this manner.

The following parameters influenced the results obtained:

- Injection process parameters (injection pressure and mold temperature). These parameters affected the conditions of filling the mold cavity and the formation of the resulting skin–core structure, which is variable along the length of the product and contributes to the heterogeneity of the mechanical properties.
- By varying the injection pressure in the range of 20–80 MPa, the mechanical properties were improved by up to 27%.
- By choosing the appropriate mold temperature (range 20–60 °C), an improvement in mechanical properties of up to 17% was found. The optimum temperature appears to be 40 °C and above.
- The change in injection pressure and mold temperature was closely related to the change in crystallinity, with changes in mechanical properties reflected in the change in crystalline phase content.
- From the results, it can be concluded that the indentation method can be used to detect changes in mechanical properties that are affected by the morphology of the polypropylene. This morphology can be correlated with the mechanical properties.
- Based on the conclusions from this work, it is possible to conclude that the properties of injection-molded products are not uniform along their length but vary locally according to the conditions in the mold. This significantly changes the view of injection-molded parts in practice, where it is not possible to consider an injection-molded product as uniform in single locations. The way in which the mold is filled plays an important role and, together with the overall flow behavior, influences the resulting properties at specific locations in the product, as well as the overall distribution of molecular orientation in the product.

## Figures and Tables

**Table 1:** BJ380MO parameters.

Properties	Value
Trade name	Borealis BJ380MO
ITT (g/10 min)	80
Density (kg/m^3^)	905
Modulus of elasticity (GPa)	1.3
Melt temperature (°C)	210–260
Mold temperature (°C)	20–60
Holding pressure (MPa)	min 20

**Table 2:** Technological parameters setting.

Technological Parameters	Value
Injection pressure (MPa)	Variations
Holding pressure	80% Injection pressure
Holding pressure duration (s)	3
Cooling time (s)	20
Mold temperatures (°C)	Variations
Melt temperatures (°C)	215
Zone n. 1 (°C)	215
Zone n. 2 (°C)	210
Zone n. 3 (°C)	205
Zone n. 4 (°C)	200
Zone n. 5 (°C)	200

**Table 3:** Varied parameter setting.

Variations of Technological Parameters	Value
Injection pressure (MPa)	20, 40, 60, 80
Mold temperature (°C)	30, 40, 50, 60
Surface of test plates (µm)	Ra 0.8

**Table 4:** Measurement parameter.

Measurement Parameter Skin and Core Layers	Value
Applied load (N)	5
Load capacity(s)	90
Loading and unloading rate (N/min)	10
Poisson’s number	0.4

## Data Availability

The original contributions presented in this study are included in the article. Further inquiries can be directed to the corresponding author.
